# A Computational Study on the Role of Parameters for Identification of Thyroid Nodules by Infrared Images (and Comparison with Real Data)

**DOI:** 10.3390/s21134459

**Published:** 2021-06-29

**Authors:** José R. González, Charbel Damião, Maira Moran, Cristina A. Pantaleão, Rubens A. Cruz, Giovanna A. Balarini, Aura Conci

**Affiliations:** 1 Institute of Computing, Fluminense Federal University, Niterói, Rio de Janeiro 24220-900, Brazil; mhernandez@id.uff.br (M.M.); aconci@ic.uff.br (A.C.); 2 Department of Internal Medicine, Fluminense Federal University, Niterói, Rio de Janeiro 24033-900, Brazil; charbeldamiao@id.uff.br (C.D.); cfontes@id.uff.br (C.A.P.); rubensacfilho@huap.uff.br (R.A.C.); giovannabalarini@id.uff.br (G.A.B.)

**Keywords:** infrared, temporal series, thermography, thyroid, cancer, numerical modeling

## Abstract

According to experts and medical literature, healthy thyroids and thyroids containing benign nodules tend to be less inflamed and less active than those with malignant nodules. It seems to be a consensus that malignant nodules have more blood veins and more blood circulation. This may be related to the maintenance of the nodule’s heat at a higher level compared with neighboring tissues. If the internal heat modifies the skin radiation, then it could be detected by infrared sensors. The goal of this work is the investigation of the factors that allow this detection, and the possible relation with any pattern referent to nodule malignancy. We aim to consider a wide range of factors, so a great number of numerical simulations of the heat transfer in the region under analysis, based on the Finite Element method, are performed to study the influence of each nodule and patient characteristics on the infrared sensor acquisition. To do so, the protocol for infrared thyroid examination used in our university’s hospital is simulated in the numerical study. This protocol presents two phases. In the first one, the body under observation is in steady state. In the second one, it is submitted to thermal stress (transient state). Both are simulated in order to verify if it is possible (by infrared sensors) to identify different behavior referent to malignant nodules. Moreover, when the simulation indicates possible important aspects, patients with and without similar characteristics are examined to confirm such influences. The results show that the tissues between skin and thyroid, as well as the nodule size, have an influence on superficial temperatures. Other thermal parameters of thyroid nodules show little influence on surface infrared emissions, for instance, those related to the vascularization of the nodule. All details of the physical parameters used in the simulations, characteristics of the real nodules and thermal examinations are publicly available, allowing these simulations to be compared with other types of heat transfer solutions and infrared examination protocols. Among the main contributions of this work, we highlight the simulation of the possible range of parameters, and definition of the simulation approach for mapping the used infrared protocol, promoting the investigation of a possible relation between the heat transfer process and the data obtained by infrared acquisitions.

## 1. Introduction

Although most thyroid nodules are benign, the possibility of malignancy should be considered. The importance of investigating thyroid nodules is related to the need of excluding the possibility of cancer, which occurs from 7% to 15% depending on patient’s age, sex, family history, and radiation exposure, among other things [1].

Benign nodules are made of mature cells, with characteristics similar to the healthy surrounding tissue [2]. These nodules present clear limits and are restricted to a mass circumscribed by a capsule or adjacent tissues. They have a slower and more organized growth than malignant nodules, and are morphologically homogeneous [3].

Malignant nodules are characterized by a disordered and uncontrolled growth of cells [4], invasion of tissues, and spreading to other parts of the body, causing metastases [5,6]. Cancerous cells divide themselves faster than normal ones and have infiltrating growth (enzymes that cause protein lysis and destruction of adjacent tissue) [7]. Cancerous cells are atypical, undifferentiated, immature, and functionally less specialized than their corresponding normal cells [8]. To grow and develop, such cells need nutrients, which causes the development of new blood vessels around them. This phenomenon increases vascularization and is named angiogenesis [9]. The nitric oxide, which is produced by cancer cells, interferes with the normal neural control of the blood vessel causing local inflammation and provides neoangiogenesis [10]. Central vascularization and a high rate of metabolic activity are important characteristics of malignant nodules [11]. Due to increased blood flow, malignant nodules could have a higher and more constant temperature than the surrounding region or benign nodules [12,13,14]. The verification (or contradiction) of these statements is the aim of this study.

Infrared Thermography (IRT) uses camera and software to convert the infrared radiation emitted by objects into temperature values [15]. Digital Infrared Thermal Imaging (DITI) [16] is a non-invasive technique based on IRT that allows to visualize and quantify temperature changes in the skin surface [17]. DITI has been successfully explored in various fields of medicine, such as the determination of circulatory problems, assessment of the body’s reaction to a medication, physiotherapy treatments, study and diagnosis of various conditions, and detection of various type of cancers [17,18,19,20,21].

Few previous works have investigated the use of IRT to aid in the diagnosis of thyroid diseases [19]. Some of them only analyze the thermal effects of hyperthyroidism and hypothyroidism by IRT [22,23]. There are works on theoretical studies of the possibility of uses IRT in thyroid nodule diagnosis [24,25,26,27,28], as well as papers evaluating the uses of IRT as a complementary technique to help on the indication of biopsy of nodules suspected of malignancy [29]. However, there is no work on simulating a complete protocol of thermography, including the transient phase, which has been considered crucial on investigating the possibility of IRT for cancer diagnosis in previous work [30] and is carried out in this article.

**Figure 1:**
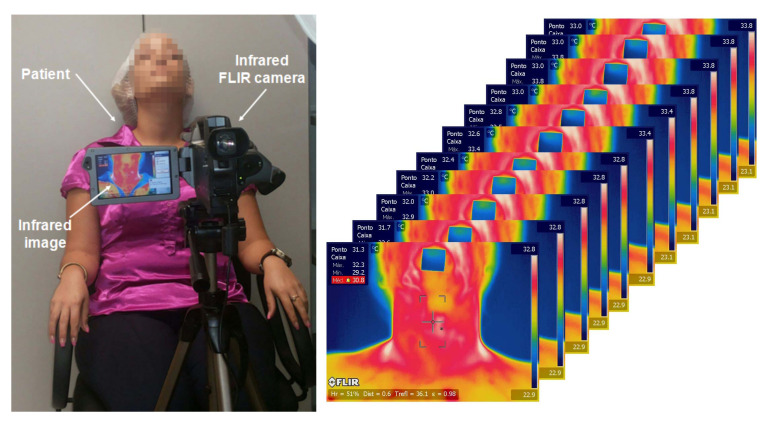
Set up of the thyroid image acquisition by infrared sensors and some images captured and stored every 5 s by DITI.

The examination room, intrinsic and extrinsic patient factors, and all details to be considered for image acquisitions, cited in [31] for medical uses of IRT were carefully considered in the simulated protocol [32]. This protocol is used to perform infrared neck examinations of volunteers with benign and malignant thyroid nodules on follow up by the medical doctors of our research group [32]. Figure 1 shows how this imaging is done, with a person in front of the camera and the sequence of images stored for each person. The work here presented is motivated by the fact that even following this protocol for all patients, the analysis of ours previous works using IRT [33,34,35] showed insufficient evidences to promote a differentiated diagnosis of the nodules. These results could be related to the influence of more factors than those previously considered [31] indicating the importance of better analysis of the problem and the here proposed simulation of patient characteristic in numerical analysis.

Clinically and biologically, the similarities or differences among nodules are evaluated by parameters completely diverse from those used in mechanical heat transfer analysis. Even the size, that is the simplest of all, has different ways of being measured: The length of the nodules in each direction, for instance, is measured by ultrasound exams [29] mainly only by the highest and smallest diameters, without relation with the body axial planes or any fixed axial reference, to allow precise positioning in the numerical model. Furthermore, thyroid nodules can appear at different positions and depths inside the gland regardless of their diagnosis [29,36].

Additionally, the vascular distribution of thyroid nodules is a very complex issue. In the literature it is common to find reports of malignant nodules “poor on the number of veins” as well as “hypervascularized benign nodules” [37,38,39]. Khadra et al. [40] mentioned that several reports have proposed that increased vascular flow may be associated with malignancy in thyroid nodules and at the same time others have described no correlation between the presence of vascular flow and risk of malignancy. They performed a meta-analysis of the literature until 2016 in order to determine whether the vascularity of a thyroid nodule can aid or not in the prediction of malignancy. Their conclusion is that there is no significant difference in vascular flow, or peripheral vascular flow rate between malignant and benign thyroid nodules. Moreover, there is no significant difference in internal vascularity between malignant and benign thyroid nodules.

Patients with thyroid nodules also have different body constitutions, summarized by the relationship between body mass (weight) and height. Therefore, all these characteristics could produce different infrared thermal patterns even in patients with the same diagnosis, suggesting that an internal heat source is dependent of the nodule echogenicity and size [41].

Other variables related to the physical phenomenon of heat transfer are: The heat rates produced by the nodules; the rates of heat produced by organs and tissues within the neck, the mechanism of heat transfer from the interior to the superficial tissues; and the anatomical and physiological characteristics of patients and nodules. All must be considered [31].

Based on the hypotheses that nodules with similar characteristics could produce similar thermal patterns, and when presenting different thermal patterns with all characteristics that are the same, could be related to nodule malignant or benign diagnostic, the objective of this work is to understand how the parameters of thyroid nodules (blood perfusion rate, metabolic heat, size, etc.) and patient features influence the identification of thyroid nodules by infrared thermography. To reach this aim two studies are performed: The first does a bio-heat transfer analysis through numerical simulations by using the COMSOL Multiphysics^®^ v5.2 [42] Finite Element (FE) software (license number: 1042008) in order to identify relevant elements and their relations. The second compares infrared examinations of volunteers presenting confirmed diagnosis with simulations to verify if the numerical results are compatible with reality detected in patients. As far as we know both studies are done for the first time to support the diagnosis of this gland by thermography.

This work is part of the research named Acquisition, storage, and verification of the feasibility of the use of thermal images in the detection of thyroid diseases (in Portuguese: “Aquisição, armazenamento e verificação da viabilidade do uso de imagens térmicas na detecção de doenças da tiróide”) designed to assess the importance of thermal imaging in the diagnostic of thyroid nodules in accompanied patients at *Antônio Pedro University Hospital* (HUAP) of the Fluminense Federal University (UFF). This research was approved by the Research Ethics Committee of the UFF, it is registered with the Certificate of Presentation for Ethical Appreciation (CAAE): 57078516.8.0000.5243, and completely detailed in the public platform of the Health Ministry of Brazil (accessible at http://plataformabrasil.saude.gov.br, accessed on 20 March 2021) [32].

**Figure 2:**
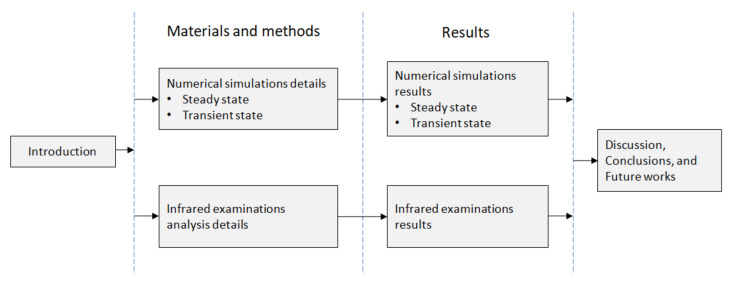
Flowchart followed in the exposition of the research.

The work is composed of five sections. The next one presents the design of the simulations and patients of the HUAP chosen and invited to be examined under the premises of the simulated protocol. Additionally the Results, Discussion, Conclusions, and Future Works sections elaborate on the subject their titles suggest. Figure 2 summarizes the work and displays the main aspect of its section contents.

## 2. Materials and Methods

All details related to the performed numerical simulations as well as infrared examinations of the volunteers are presented in the next subsections.

### 2.1. Details of the Bioheat Numerical Analysis

This section shows theoretical and practical details about the bioheat transfer analysis performed by using the COMSOL Multiphysics software [42]. Two types of numerical studies were carried out to evaluate how and which parameters can influence the bioheat transfer related to a thyroid nodule: One considers the skin in thermal equilibrium with the environment, and the other includes a forced initial ventilation.

The principal stages required to perform the Finite Element numerical simulations includes: The creation of a simplified neck geometry considering the most important organs and tissue layers (and their variations as nodule size and fat thickness), selection of the mathematical model for bioheat transfer and boundary conditions according to the problem, definitions of thermophysical parameters for each organs and tissue, construction of a convergent mesh representing the geometry, and computation of a solution for each specific configuration of geometry and parameters. These stages are later detailed.

#### 2.1.1. Neck Simplified Geometry

**Figure 3:**
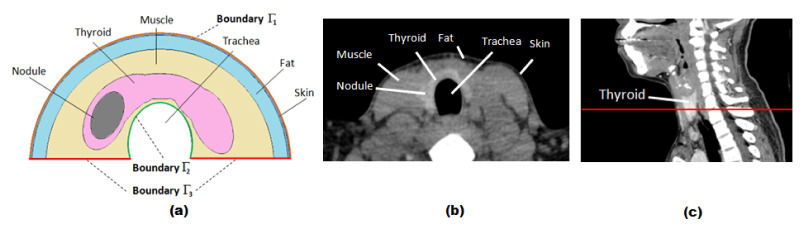
Simplified 2D geometry used in the FE simulations (**a**) based on the best CT view of the thyroid gland (**b**) that is the cross section of the neck represented by the red line of the sagittal CT slice on (**c**).

The 2D simplified geometry of the neck presented in Figure 3a was used for the basic model. Although this is a 3D problem, it is observed that there is no heat exchange along the height of human beings. Therefore, it is suitable for the study of heat transfer to assume a thermal continuity in this direction. Hence, we simplify the study by analyzing the 2D elements presenting great influence of this region in the best direction for such analysis, that is the cross section (Figure 3b) of the neck represented by the red line on Figure 3c.

The simulated neck considers skin, fat, and muscle layers, the thyroid gland, and an elliptic nodule inside the thyroid lobe. The thicknesses of the skin and muscle tissues in Figure 3a are 0.1 cm and 1.0 cm [43], respectively. The thickness of the fat layer and the nodule size will be modified in the experiments to study their influence on the skin temperature. Simple geometric elements (cylinders and ellipses) and logical operations (union and difference) available in COMSOL Multiphysics software were used to create this geometry [44]. The effects of the trachea, arteries, and jugular veins of the neck were disregarded. The simplified geometry was modeled by an extremely-fine triangular mesh using a proper tool of the used software, being its convergence tested in a preparatory stage of this research [30].

#### 2.1.2. Mathematical and Physical Model

The Pennes’ bio-heat transfer model is used in the simulation [45]. This model considers the total energy balance and its storage, the internal energy rate, heat conduction, convection inside and outside the body and environment, as well as local heat generation [15]. At the same time, chemical and electrical effects are neglected in the model [41]. Pennes’ model uses the modified transient heat conduction equation and two heat sources, both per unit of time and volume: One due to the metabolic effect, and the other due to the energy exchange between tissue and blood [46]. An external source could be included but it does not match the protocol of exams here considered [47,48].

Simulations consider no skin ventilation, after superficial cooling, and then reheating [30]. The cooling period simulates the direct neck exposition to the electric fan airflow of the examination protocol. The reheating period is based on the return to thermal equilibrium with the environment (the examination room temperature is 25 ${}^{{}^{\circ}}$C) immediately after the cooling period.

Simulations at the transient state attempt to reproduce the protocol for thyroid infrared examinations used in our university’s hospital [32]. In this exam, the patient the neck surface is cooled with an airflow, reaching 30 ${}^{{}^{\circ}}$C or over five minutes under room temperature. Then the airflow is suspended and one thermogram is captured every 15 s during reheating for five minutes [47,48]. This procedure is based on Dynamic Infrared Thermal Imaging (DYTI), as also used in [21].

- Formulation and conditions without skin ventilation.

The mathematical formulation of the Pennes’ model at steady state is (1):

(1) $$0=\nabla \left[k\left(x,y\right)\nabla T\left(x,y\right)\right]+w_{b}\rho_{b}c_{b}\left(x,y\right)\left[T_{b}-T\left(x,y\right)\right]+Q_{m}\left(x,y\right).$$

In Equation (1), *k* is the thermal conductivity, $w_{b}$ is the blood perfusion (blood flow rate per volume unit of tissue), $\rho_{b}$ is the blood mass per volume (or density), $c_{b}$ is the blood specific heat, $T_{b}$ is the arterial blood temperature at the capillary level, and $Q_{m}$ is the metabolic heat of the tissue.

The boundary conditions are: The thermal convection (2) [49] with the environment at skin surface $\Gamma_{1}$ (orange line in Figure 3a), thermal insulation (3) in the trachea boundary surface $\Gamma_{2}$ (green line in Figure 3a), and prescribed temperature (4) at $T_{p}=37{\;}^{{}^{\circ}}$C on the remaining surfaces $\Gamma_{3}$ (red lines in Figure 3a):

(2) $$\left(x,y\right)\epsilon \Gamma_{1}:-n\cdot k\left(x,y\right)\nabla T\left(x,y\right)=h\left[T_{air}-T\left(x,y\right)\right]$$

(3) $$\left(x,y\right)\epsilon \Gamma_{2}:-n\cdot k\left(x,y\right)\nabla T\left(x,y\right)=0$$

(4) $$\left(x,y\right)\epsilon \Gamma_{3}:-n\cdot k\left(x,y\right)\nabla T\left(x,y\right)=37{\;}^{{}^{\circ}}C.$$

In Equation (2) we use for the convection coefficient, h=10 W/(m${}^{2}$K), a typical value for natural convection and, for air temperature that of the room ($T_{air}=25{\;}^{{}^{\circ}}$C) [25,26,28].

- Formulation and conditions for cooling and reheating.

Pennes’ model at transient state is represented by (5). In this equation, *p* and *c* represent the specific mass and specific heat of tissue, respectively. The other parameters were already commented in Equation (1):

(5) $$\rho \left(x,y\right)c\left(x,y\right)\frac{\partial T\left(x,y,t\right)}{\partial t}=\nabla \left[k\left(x,y\right)\nabla T\left(x,y,t\right)\right]+w_{b}\rho_{c}c_{b}\left(x,y\right)\left[T_{b}-T\left(x,y,t\right)\right]+Q_{m}\left(x,y,t\right).$$

To simulate the transient thermal stress due to cooling, Equation (2) representing the convection in the skin surface ($\Gamma_{1}$), is also considered. However, in this case, a convection coefficient equal to h=50 W/(m${}^{2}$K) is used to simulate forced thermal convection due the airflow [50]. The air temperature is held in $T_{air}=25{\;}^{{}^{\circ}}$C because the fan airflow hits the neck with pressure and speed, but with this temperature (according previous experiments performed that consider temperatures on the skin’s surface after five minutes of cooling in real examinations).

Thermal insulation (3) in the trachea boundary surface ($\Gamma_{2}$), and prescribed temperature (4) at $T_{p}=37{\;}^{{}^{\circ}}$C on the remaining surfaces ($\Gamma_{3}$), were also considered as boundary conditions. The solution obtained at the steady state was used as the initial condition for the cooling period. For this reason, each curve on the graphs in the session of “heat transfer analysis at transient state” begins differently related to their temperature.

To simulate the thermal reheating after the cooling period, thermal convection (2) in the skin surface ($\Gamma_{1}$) was considered again. In this case, the convection coefficient (h=10 W/(m${}^{2}$K)) and air temperature ($T_{air}=25{\;}^{{}^{\circ}}$C) were used to simulate the natural thermal convection with the environment. Thermal insulation (3) in the trachea boundary surface ($\Gamma_{2}$), and prescribed temperature (4) $T_{p}=37{\;}^{{}^{\circ}}$C on the remaining surfaces ($\Gamma_{3}$), were also considered. The last solution obtained in the cooling period was used as the initial condition for the reheating period.

#### 2.1.3. Thermophysical Parameters Used in Experiments

Table 1 presents the values of thermophysical parameters for every simulated tissue [26,28,51]. The $\rho_{b}$ and $c_{b}$ parameters are considered to be equal to specific mass (ρ) and specific heat (*c*) of the tissues.

#### 2.1.4. Details of Steady State Experiments

Four aspects are considered in steady state experiments: Two are related to size and two represent heat transfer parameters.

**Figure 4:**
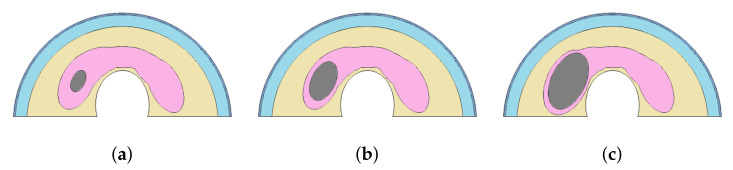
The diameters of nodules used in the numerical simulations: (0.7; 1.2 cm) small (**a**), (2.2; 1.26 cm) medium (**b**), and (3.14; 1.80 cm) large (**c**).

**Figure 5:**
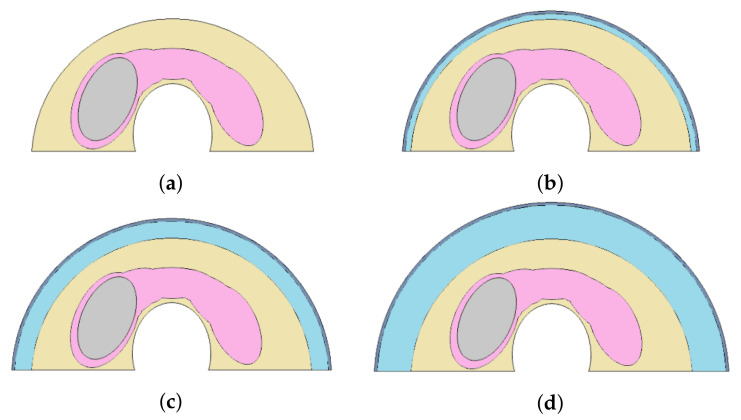
Fat tissue thickness used in the simulations: 0 cm (**a**), 0.3 cm (**b**), 0.6 cm (**c**), and 1.2 cm (**d**)

The size-related experiment considers nodule and fat sizes. The fat tissue layer varies among individuals. Fat thickness is associated with the Body Mass Index (BMI) and can be measured by Ultrasound (US) examinations [37,38]. Three sizes of nodules, with the diameters shown in Figure 4 and four fat layers of thickness (0, 0.3, 0.6, and 1.2 cm) were considered (as shown in Figure 5).

As mentioned before, the metabolic heat ($Q_{m}$) and blood perfusion rate ($w_{b}$) of a thyroid nodule are complex to be estimated [52]. We did not find reports that mention these features for benign or malignant nodules up until now. In case of a malignant thyroid nodule, Bittencourt et al. [26] adopt values of $Q_{m}=$ 42,000 W/m${}^{3}$ and $w_{b}=0.465$ (mL/s)/mL using other malignant tumors as a reference. For normal thyroid tissue, the values of these parameters can be found as $Q_{m}$ = 4200 W/m${}^{3}$ and $w_{b}=0.098$ (mL/s)/mL [26,28,51]. Therefore, variations of these characteristics must be consistent with benign or malignant types of nodules because they are the parameters that allow differential behaviors compatible with vascularization and heat production, such as described by some works in the medical area [53,54]. Table 2 shows the possible values of the metabolic heat and blood perfusion rate that are used. The lower values of each parameter in this table are that of normal thyroid tissue. The other two are the same of [26].

#### 2.1.5. Details of Experiments at Transient State

As commented before, to simulate the image acquisition protocol, all studies at transient state were done in three phases: (1) The problem at steady state was resolved, (2) a cooling period during a specific time is simulated using the solution of the first phase as the initial condition, and (3) the reheating period is also simulated during a specific time using the last solution of the cooling period as the initial condition.

Each possible element that can present influences on the heat transfer from the nodule to the skin surface were evaluated by the same software used previously. They are: (1) Four fat tissue thickness (0, 0.3, 0.6, and 1.2 cm), (2) variations of nodule diameters from (0.7; 1.2 cm) small and (2.2; 1.26 cm) medium to (3.14; 1.80 cm) large, (3) the metabolic heat, and (4) blood perfusion of the nodule. The same parameters were used in the simulations at steady state were used in the transient state.

For each case, temperature series were constructed with temperature values taken at every 15 s during the cooling and reheating periods. In some cases, symmetrical points, concerning the body axial axis, are investigated, as well. Temperature series were also obtained from a fixed point located in the skin surface in the front of the nodule, in each simulation.

### 2.2. Details for Parameter Analysis Using Infrared Examinations of the Volunteers

Data from eight volunteers, patients of the (HUAP/UFF) University Hospital [55], were selected to verify and compare real data with the numerical findings, and to show the influence of the nodules and patient parameters. Solid thyroid nodules were encountered in each of them and they were examined by using the same image acquisition protocol [32].

The used protocol establishes orientations in all levels (i.e., from patient’s orientation to technical and environmental requirements) and the methodology for the exam based on Dynamic Infrared Thermography (DYTI) to evaluate the temperatures on the neck’s surface during the return to thermal equilibrium after thermal stress.

The thermal stress consists of neck cooling by an airflow (made by an electric fan) until it reaches a mean value of 30 ${}^{{}^{\circ}}$C or for 5 min, as the maximum cooling time [47,48]. After thermal stress, 20 infrared images are acquired, every 15 s for 5 min. An additional image is captured to indicate the position of the nodules with an insulating thermal material (that is fixed in the patients by their accompanying medical doctor after the identification of nodular position by touch or based on previous ultrasound examinations). All infrared examinations were performed using an FLIR infrared camera model SC620. The camera sensor has a NETD (Noise Equivalent Temperature Difference) value less than 40 mK, capture range of −40 ${}^{{}^{\circ}}$C to 2000 ${}^{{}^{\circ}}$C, and produce thermogram up to 640 × 480 pixels.

**Figure 6:**
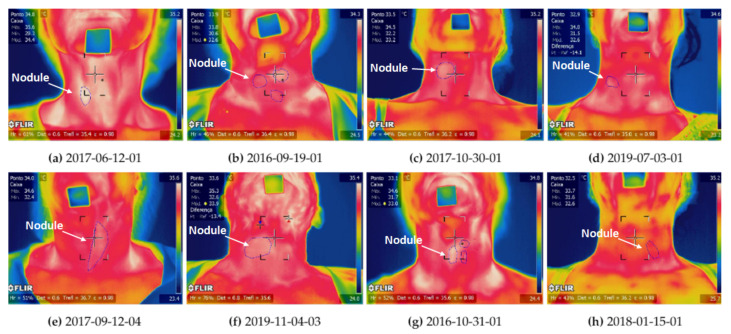
The last thermogram of eight patients, where it is possible to see the position and size of the nodules marked with a fine line by endocrinologists. Details of each person are in Table 3.

Figure 6 shows the last thermogram of the infrared examinations of the eight patients. The region of nodules was marked with a thin blue line in each thermogram, by the medical doctor (M.D.) responsible for the patient and then confirmed by the other M.D. based on previous exams.

Table 3 condenses all relevant data from nodules and patient bodies: The diameters (cm; cm); the principal ultrasound characteristics defined in columns 3 to 6 as Yes or No and related to the presence of: Micro-calcification, complete halo, regular contours, heterogeneity, and the Chammas vascularization pattern in each nodule; the cytopathological report (using the Bethesda classification); the average body temperature (${}^{{}^{\circ}}$C) measured by an armpit thermometer; the mass of the patient body (kg), the patient height (cm), the patient body mass index (BMI) (kg/m${}^{2}$); and the confirmed (by cytology) diagnostic of the nodule (B—benign and M—malignant).

- Influence of fat tissue thickness.

To show the influence of fat tissue thickness using real data, two volunteers were selected. They are the patients identified as 2017-06-12-01 and 2016-09-19-01 in the database and can be seen in Figure 6a,b.

The nodules of both patients have similar dimensions, the same pattern of vascularization (the third level in the Chammas scale [56]), and the same risk of malignancy in the cytopathological examination (6), as well. However, these patients present opposite BMI (and so fat tissue layers) as can be seen in the penultimate column of Table 3.

- Influence of nodule size.

To show the influence of nodule size using real cases, the patients 2017-10-30-01 (Figure 6c) and 2019-07-03-01 (Figure 6d) of the database were selected. Their nodules have similar ultrasound characteristics and pattern of vascularization, but different dimensions, i.e., the nodule diameters of these patients are (4.2; 2.3 cm) and (1.4; 1.0 cm), respectively As can be seen in the second column of Table 3.

- Influence of vascularization.

To compare the influence of vascularization of real data and simulations, two pairs of patients were selected. The first pair is composed by patients 2017-09-12-04 (Figure 6e) and 2019-11-04-03 (Figure 6f). Their malignant nodules have similar dimensions and ultrasound characteristics, but a different pattern of vascularization (2 and 5 in the Chammas scale).

The second pair (2016-10-31-01 (Figure 6g) and 2018-01-15-01 (Figure 6h)) presents nodules with opposite diagnoses. The patient 2016-10-31-01 has three benignant thyroid nodules, but only the biggest (the first from left to right in Figure 6g) was considered. The patient 2018-01-15-01 has a malignant thyroid nodule. Their nodules have similar ultrasound characteristics, as shown in Table 3 columns 4 to 6. Both patients are also skinny (i.e., similar body mass index), decreasing a possible influence of fat tissue. Moreover, their nodules have similar peripheral vascularization (second and third levels in the Chammas scale [56]).

## 3. Results

The thermal energy transfer occurs from a high temperature place to a lower temperature location and may occur under steady or unsteady state conditions. Under steady state conditions, the temperature within the system does not change with time. When the temperature is time related, the system is considered under unsteady state conditions. Both states are analyzed in the experiments of this work.

### 3.1. Bio-Heat Transfer Analysis at Steady State

This subsection presents the results of simulating the bio-heat transfer when the object under study is in equilibrium with the environment. Additionally, it shows how the body anatomy and tumor characteristics (specifically the nodule size and fat tissue thickness) could influence the temperature transmission up to the skin.

#### 3.1.1. Variation of Temperatures from Inside the Body to the Neck Surface

**Figure 7:**
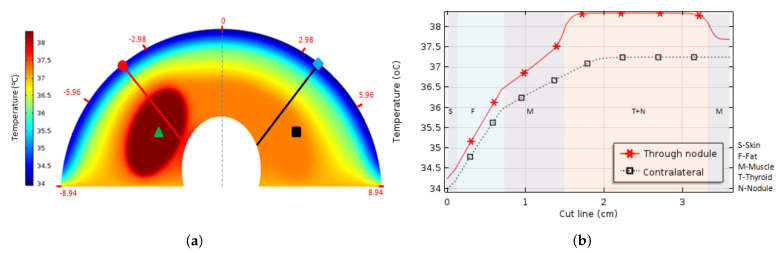
Temperature variations inside the neck represented in colors (**a**) and graphically along symmetrical lines (**b**). They are computed by finite element simulation considering a big elliptic nodule (17.76 cm^2^) and a medium fat tissue layer (thickness of 0.6 cm). Both (red and black) lines represent the temperature going from the surface to the trachea: They cross the skin (S), fat (F), muscle (M), and thyroid (T) layers represented by the smooth colors. The red line goes through the nodule (N) as well.

Figure 7a shows the temperature variations represented by the color scale in the right (going from navy-blue = 34.0 ${}^{{}^{\circ}}$C to dark-red = 38.5 ${}^{{}^{\circ}}$C), computed by one of the several numerical simulations done when an elliptic nodule (with diameters of 3.14 and 1.80 cm) considered inside a thyroid (in a neck with fat tissue layer equals to the human average value, which has a thickness of 0.6 cm for the frontal region). There are two lines and four points that must be noted in Figure 7a.

The red line crosses the nodule and the black is symmetrical to the red considering the middle of the image. The point represented by the green triangle is the nodule center, and the red circle point at the beginning of the red line its the location on the skin and zero horizontal coordinates of Figure 7b. The black square and cyan diamond are symmetrical to the green triangle and red circle respectively. The red numbers in the perimeter of Figure 7a represent the distance of the neck position surface to the symmetrical axis (in the front of the neck). In other words, the red numbers in Figure 7a indicate the length of the arc at the skin surface (for a neck with a perimeter of 36 cm approximately) when a thickness equals to 0.6 cm is considered for the fat tissue layer.

The temperature variations presented in Figure 7b, from the skin to the trachea, are computed by the numerical simulation along counter side lines considering the sagittal plane. The red line crosses the nodule and the black line (symmetrically positioned to the red one) crosses only the healthy thyroid lobe. As can be seen, the temperature variation into the fat layer for both lines was approximately equal to $△T_{F}$ = 2.0 ${}^{{}^{\circ}}$C showing the great importance of the fat tissue on the isolation of the internal body temperature from the superficial temperature.

It is interesting to note that temperatures of approximately 37.2 ${}^{{}^{\circ}}$C were obtained in the right thyroid lobe region (black square position), which is the region without a nodule corresponding to normal internal body temperature. Temperatures around 38.3 ${}^{{}^{\circ}}$C were found in the central region of the nodule (in the green triangle position).

The simulation output temperatures of approximately 34.3 ${}^{{}^{\circ}}$C in the neck surface related to the thyroid nodule region (around the red circle), and 34.0 ${}^{{}^{\circ}}$C in the counter side region, that is the symmetric point in respect to the dashed line in the figure that represents the human sagittal plane (the point marked with a cyan diamond). This indicates a temperature variation of approximately $△T_{CL}$ = 0.3 ${}^{{}^{\circ}}$C in the neck surface caused by the presence of the nodule when compared with the healthy region (for this specified nodule size and fat thickness).

#### 3.1.2. Influence of Nodule Size and Fat Tissue Thickness in the Surface Temperature

**Figure 8:**
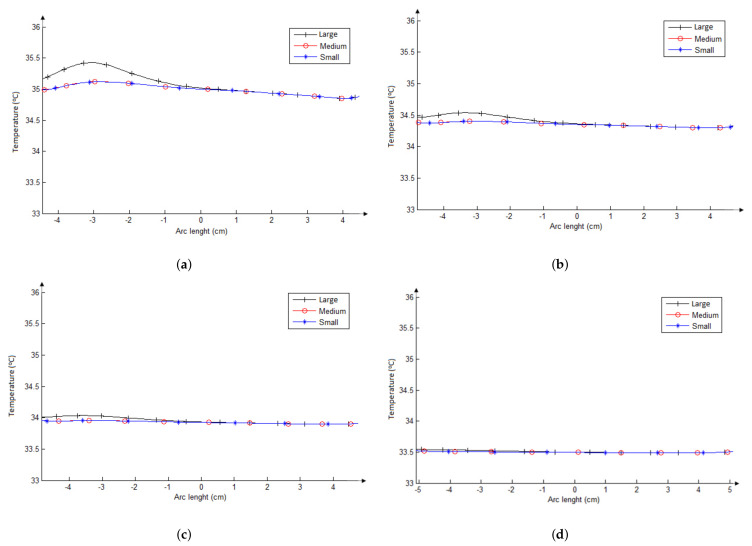
Superficial temperatures for the large (black line), medium (red line), and small (blue line) nodules of necks with fat tissue layer thickness from 0 cm (**a**), 0.3 cm (**b**), 0.6 cm (**c**), to 1.2 cm (**d**).

There are four graphs in Figure 8, each one with three curves showing the thermal distribution on the skin’s surface of the neck obtained from the simulations of nodules with three sizes (black = Large, red = Medium, and blue = Small). In the graphs four fat tissue thickness are considered: 0 cm (Figure 8a), 0.3 cm (Figure 8b), 0.6 cm (Figure 8c), and 1.2 cm (Figure 8d). For these graphs, the central position (0 cm in the horizontal axis) is related to the dashed line in Figure 7a that represents the human sagittal plane and the negative and positive values in the horizontal axis are related to the left and right part from the dashed line, respectively. In these simulation, the parameters used for metabolic heat is $Q_{m}$ = 42,000 W/m${}^{3}$ and the blood perfusion rate is $w_{b}=0.465$ (mL/s)/mL [26]. These values can be representative of malignant nodules.

As can be seen, the thermal influence of the nodules on the skin’s surface decreases as the thickness of the fat layer increases. There is a perceptible surface temperature variation between the point just in front of the nodule (around −3 cm of the horizontal axis of the graphs, that represent the neck superficial position) and its counter side point (around 3 cm of the horizontal axis of the graphs) for the simulation using the thinnest fat layer (Figure 8a). However, this variation (between symmetric points) is almost imperceptible when the fat thickness increases, as can be seen in the other graphs of Figure 8. There is not a nodule significant effect for the fat layer with a 1.2-cm thickness. Moreover, temperature variation between symmetrical superficial points is proportional to the size of the nodules (i.e., increase when the area of the nodules increases). Note that the fat layer can change the superficial temperature maximally 2 ${}^{{}^{\circ}}$C and the nodule size 0.6 ${}^{{}^{\circ}}$C (for the same neck). Therefore, the simulations presented in Figure 8 show that the thermal insulation effect of the fat tissue layer is much more important than the nodule size, although both present the expected influence on the surface temperature.

#### 3.1.3. Influence of the Metabolic Heat and the Blood Perfusion Rate in the Neck Heat Transfer

**Figure 9:**
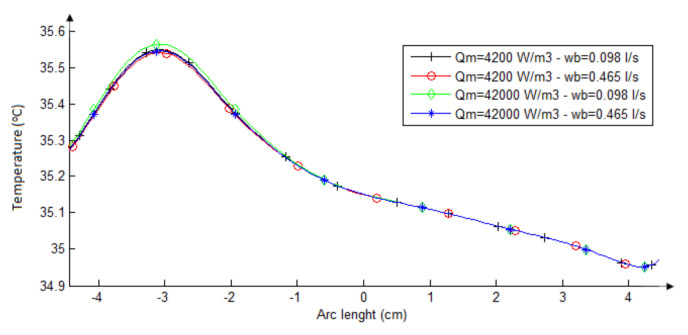
Surface temperatures obtained from the four possible combinations of metabolic heat and blood perfusion values considering no fat tissue and the biggest thyroid nodule.

Table 2 shows the four possible combinations from values of metabolic heat and blood perfusion rate obtained from the literature for normal thyroid (second row) and malignant tumor tissues (fifth row of the table). Curves in Figure 9 shows the thermal distribution on the neck surface computed considering all combinations of the values of metabolic heat and blood perfusion for the thyroid nodule. In these simulations, the best possible conditions of the other parameters for skin temperature difference observation between counter side points were used (that is, the biggest nodule with diameters of 3.14 and 1.80 cm and smaller possible fat tissue thickness: 0 cm). Although it is very difficult to find a correct relation among heat transfer parameters and biomedical behavior, the simulations presented in Figure 9 shows that for any combination that could be representative of malignant or benign nodules, it is not possible to detect different behaviors among these curves. All combinations promoted the same modification on the skin’s surface between counter side positions of 0.5 ${}^{{}^{\circ}}$C.

### 3.2. Bioheat Transfer Analysis at Transient State

This subsection shows the results of the performed simulations at the transient state. In the patient’s examinations reported in the next subsection that such a condition is induced.

#### 3.2.1. Influence of Fat Tissue Thickness and Nodule Size

**Figure 10:**
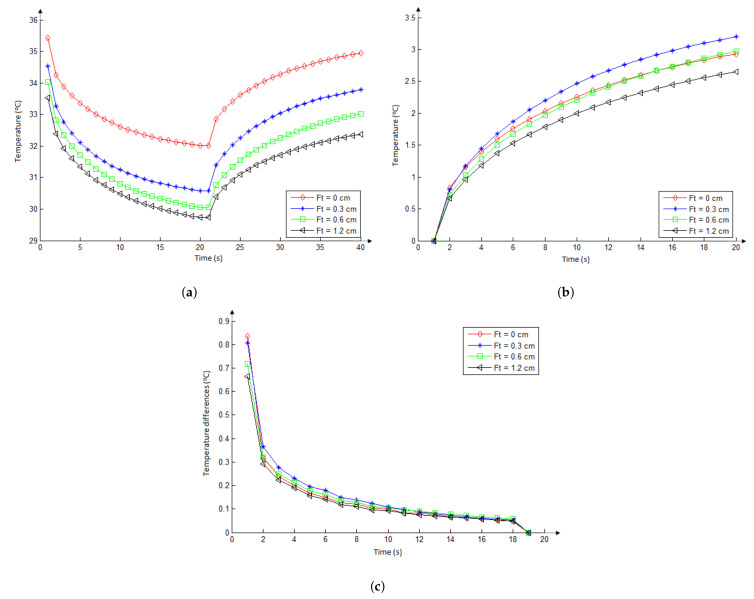
Influence of fat tissue thickness on the skin temperature just in front of the nodule (the red point in Figure 7) over the time of the exam of the simulated protocol (**a**). Temperatures at the same position on the natural reheating stage (**b**) but considering each curve starting with the same temperature for better comparison. The rate of superficial temperature at same point (**c**). (Note the peculiar behavior of the zero value differing from the tendency to zero of 0.6 and 0.3 cm in (**b**) and (**c**)).

The four curves in the graph of Figure 10a show the temperature series related to the red circle at the beginning of the red line in Figure 7a that is located in the skin surface, computed by the four fat tissue thickness considered (0, 3, 6, and 12 cm). These results were obtained using the biggest nodule (with diameters of 3.14 and 1.80 cm or area of 17.76 cm${}^{2}$), the metabolic heat of $Q_{m}$ = 42,000 W/m${}^{3}$, and blood perfusion rate of $w_{b}$ = 0.465 (mL/s)/mL for the nodule. In the same way as in the steady state, the lower temperature variations (curve with black-triangle details) were obtained in the skin surface when the major fat tissue thickness was considered, showing that this layer reduces the influence of internal conditions in the neck surface temperature.

Figure 10a shows that the temperature limits for the curve representing the biggest fat tissue (1.2 cm) are: 33.6, 29.7, and 32.4 ${}^{{}^{\circ}}$C. These limits for the opposite case, the numeric abstraction representing a tendency of the fat layer to be on the lower possible case, i.e., having no fat tissue (0 cm), are 35.5, 32.3, and 35.3 ${}^{{}^{\circ}}$C. For others (i.e., fat thickness between these values), the curves present limits almost visually proportional. Such variations are around 4 ${}^{{}^{\circ}}$C in cooling and 3 ${}^{{}^{\circ}}$C on warming. Note that they are greater than any other variation in the steady state (even due to the size of the nodule) showing the importance of inducing a dynamic examination in this area of the human body for all types of patients under study. The thermal insulation is proportional to the thickness of the fat layer.

The curves in Figure 10b show only the reheating period of the previous temperature series (due to the infrared examinations of patients be performed in the reheating period) and related to its first value (i.e., they show the temperature series starting with the same value in order to promote a better form of representation for the total variation of each one). As can be seen, temperatures related to the fat tissue layer of 1.2 cm (curve with black-triangle details) are smaller than those related to the fat tissue layer of 0.6 cm (curve with green-square details), and such temperatures are smaller than those related to the fat tissue layer of 0.3 cm (curve with blue-plus details) as well. The position of the null thickness curve (red one) is not representative because this supposition of a no-fat layer must be seen as a tendency and not something real (in itself). In other words, its position in this graph may be a numerical inconsistency related to the abstraction realized by such an initial premise.

The curves in Figure 10c show the difference between two consecutive temperatures ($\Delta_{j,i}=S_{j,i}-S_{j,i-1}$) in each of the temperature series ($S_{j}$) starting in the reheating period. As the difference between each respective time is constant, these curves can be directly associated to the rate of temperature changes. The higher heating rate appears for the thinner layer of fat (curve with blue-plus details related to 0.3 cm). By comparing the other two thicker layers, the speed of temperature change is proportional to the reduction of the fat layer. Or the speed of temperature change is inversely proportional to the thickness of the fat layer (again the relative position of the curve related to the null thickness is not representative).

**Figure 11:**
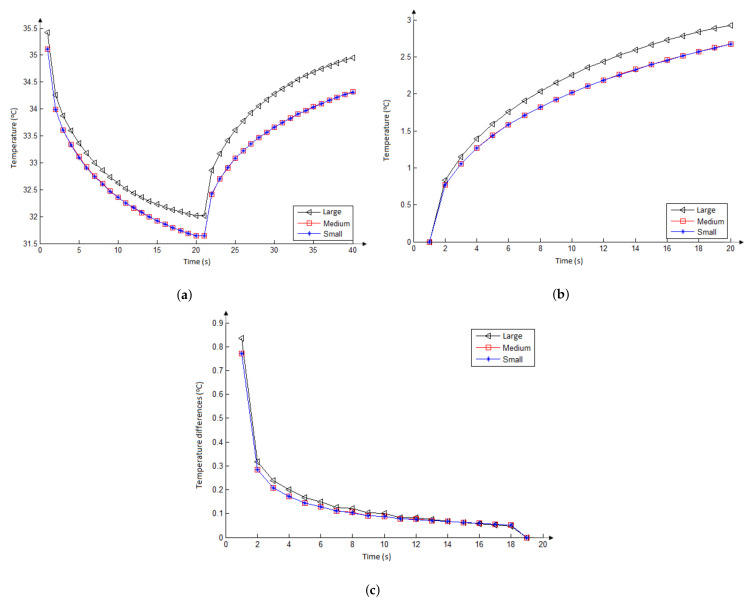
Influence of nodule size in the superficial temperature in front of the nodule considering: All the time of examination in the simulated protocol (**a**) and the natural reheating stage (**b**), where each curve was represented starting with the same temperature for better comparison. Influence of nodule size in the velocity of superficial temperature change, this velocity is related to the differences between two consecutive temperatures (**c**).

The three curves in the graph of Figure 11a show the temperature series related to the same point (marked by the red circle in Figure 7a) located on the skin’s surface considering three nodule sizes: Large (diameters of 3.14 and 1.80 cm, area = 17.76 cm${}^{2}$); medium (diameters: 2.2; 1.26 cm, area = 8.71 cm${}^{2}$); and small (diameters: 1.2, 0.7 cm, area = 2.64 cm${}^{2}$).

The curves in Figure 11b also show the reheating period of these three temperature series related to the first value of each of them. These results were obtained using the smaller possible fat layer (fat thickness = 0 cm), metabolic heat of $Q_{m}=$ 42,000 W/m${}^{3}$, and blood perfusion rate of $w_{b}=0.465$ (mL/s)/mL for the nodules. They show that the larger nodule presents the highest temperature (curve with black-triangle details) variations. No differences were found between the temperatures of the two smaller nodules. This analysis shows that larger nodules present more influence on the skin surface in the transient examination as occurs in the steady state study.

Figure 11c show the temperature differences between two consecutive temperature values ($\Delta_{j,i}=S_{j,i}-S_{j,i-1}$) in each of these three temperature series ($S_{j}$) starting in the reheating period. The curves show that the nodules with the same parameters and different sizes did not show important differences in the rate of heating on the skin’s surface.

It is important to note that the final temperature in the reheating stage (in all the graphs of these studies) is less than the initial temperatures at the start of the cooling stage for all fat tissue thickness and nodule sizes considered. This shows that five minutes for the reheating stage is not sufficient to reach the same initial temperatures. The temperature before the cooling stage will only be reached in more time than that suggested by the acquisition protocol.

#### 3.2.2. Influence of Metabolic Heat and Blood Perfusion Rate

**Figure 12:**
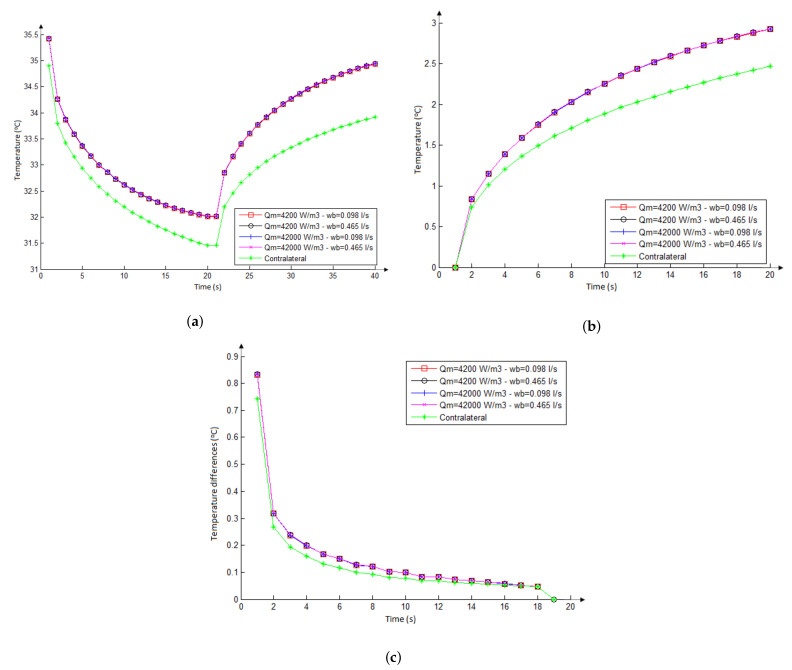
Influence of the metabolic heat and blood perfusion rate at transient state for possible combinations of nodular properties in front of it and on the symmetric neck position (on the red circle and cyan diamond of Figure 7a). Considering all simulation time (**a**). The reheating stage considering all curves starting at the same temperature (**b**). The difference between consecutive temperatures, representing the heating rate (**c**).

Temperature series in Figure 12 were performed considering the larger nodule (diameters equal to 3.14 and 1.80 cm) and no fat layer (i.e., a thickness of $F_{t}$ = 0 cm). The three graphs were obtained considering all combinations of metabolic heat and blood perfusion (shown in Table 2). As can be seen, no differences were obtained for any combinations of these parameters for the nodule. This means that whatever could be the combination of them, within a range found in the literature, that represent a malignant or benign behavior, it is impossible to detect skin surface difference on temperature related to them.

However, it can be seen that there is a difference between the series of temperatures related to the placed on the skin in front of the nodule and its counter side point (marked with the red circle and cyan diamond in Figure 7a). This means that it could be possible to distinguish if there is a nodule (at least for big nodules and skinny neck). Moreover, this difference is greater just after the end of the refrigeration process showing the importance of the examination under the induced change of temperature.

### 3.3. Results of Infrared Examinations

This subsection shows the acquired temperatures in the exams of eight volunteers (patients on follow up in the university hospital) that are examined by using the protocol simulated by the numerical study. Several other details from previous exams, performed during their follow-up, are known (Table 3). The patients are selected when their exams and data better match the hypotheses considered in the numerical simulations.

**Figure 13:**
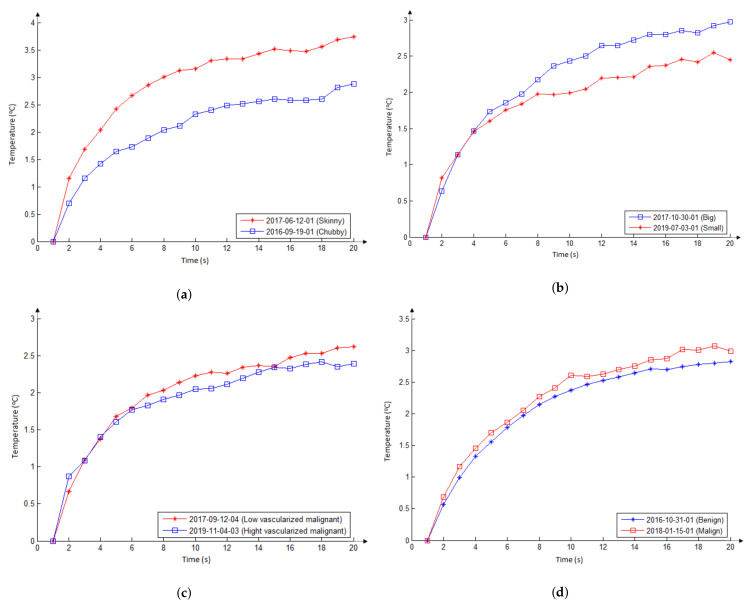
Temperature series in front of eight nodules of patients of Figure 6 obtained by the infrared imaging comparing: two benign nodules from two patients with different BMI indexes (**a**), two malignant nodules with different sizes (**b**), two malignant nodules with different pattern of vascularization (**c**), and a benign with a malignant nodule (**d**) as commented in Section 3.3.

A temperature series was organized from the infrared examinations of each patient. For it, firstly a point of the central nodule region (manually indicated by a doctor on the thermogram) was considered. Then, this location was used to compute the average temperature of an 11×11 window around it for each thermogram. The combination of them represents the patient. When two patients are compared, their series are redrawn from the first value to show them starting at the same point, as shown in Figure 13.

#### 3.3.1. Influence of Fat Tissue Thickness

To analyze the influence of the fat tissue thickness on temperature series, the infrared examinations of the patient 2017-06-12-01 and 2016-09-19-01 were compared in this subsection (see them at Figure 6a,b).

Curves in Figure 13a show their temperature series (related to the patient initial temperature, as commented in Section 3.3). The series of the chubby patient (blue line) is below that of the skinny patient (red line) for the reheating stage. This shows the same behavior related to the thermal insulating effect of the fat tissue layer presented in the numerical modeling (Figure 10b).

#### 3.3.2. Influence of Nodule Size

To analyze the influence of nodule size, we chose two patients presenting almost the same other characteristics except their nodular size. For this, the patient 2017-10-30-01 and 2019-07-03-01 were selected to have their series compared. These patients have a unitary malignant thyroid nodule with a similar pattern of vascularization and ultrasound characteristics (Table 3). Figure 6c,d present them.

The curves in Figure 13b show the temperature in relation to the initial temperature of the patient. As can be seen, the series of the patient with the biggest nodule (blue line) has values greater than those of the patient with the smaller nodule (red line). It means that the bigger nodule has more influence on temperatures of skin surface than the smaller one, as shown in the numerical simulations (Figure 11b).

#### 3.3.3. Influence of Vascularization

Two studies related to the influence of vascularization are presented in this subsection. Firstly, two patients with malignant thyroid nodules but a low and high vascularized nodules are compared. Then, the other two with a similar pattern of vascularization but presenting a malignant and benignant nodule are confronted.

The patients 2017-09-12-04 and 2019-11-04-03 were selected for the first comparison. The images in Figure 6e,f present them. They have a unitary malignant thyroid nodule with similar dimensions and different patterns of vascularization. Both are skinny, having a similar body mass index. The nodule of the patient 2017-09-12-04 has only peripheral vascularization (second level in the Chammas scale, Ref. [56]) and the nodule of the patient 2019-11-04-03 has predominant central vascularization (fifth level in the Chammas scale) (Table 3).

Curves in Figure 13c show temperatures concerning the first value obtained from the infrared examinations. As can be seen, both series are very similar. This suggests that the difference of vascularization patterns of these nodules (with the same diagnostic, similar dimensions, and ultrasonographic characteristics) causes little or no difference in the skin surface during examination with infrared sensors. This fact was also observed in the numerical simulations (Figure 12).

The patients 2016-10-31-01 and 2018-01-15-01 were selected for the second comparison. They have thyroid nodules with different diagnoses (Table 3) but nodules with similar dimensions and ultrasound characteristics, as shown in Table 3. In Figure 6g,h, patient 2016-10-31-01, who has three benignant thyroid nodules, but only the biggest (the first from left to right in Figure 6g) was compared. Patient 2018-01-15-01 has one malignant thyroid nodule. These nodules have peripheral vascularization (second and third levels in the Chammas scale [56], for patients 2016-10-31-01 and 2018-01-15-01, respectively).

Curves in Figure 13d show the series concerning these nodules obtained from the infrared examinations. As can be seen, both are very similar. These two nodules with similar characteristics and similar patient’s characteristics cause a similar thermal effect, even with different diagnoses. This fact was also observed in the numerical simulations of Figure 9. Then, the results of this subsection suggest that the thermal effects of thyroid nodules on the neck surface are not sufficient to differentiate benign from malignant, and for this other features or methods must be considered.

## 4. Discussion

The heat produced by thyroid nodules (malignant or benignant ones) could constitute visible patterns on the skin when acquired by infrared sensors under specific conditions [41,57]. However, to understand the influence of all related parameters a numerical analysis must be done because, due to their diversity, each possible combination could not be easily found in human beings for the identification of their influence on the skin’s temperature. Therefore, a parametric study is paramount to understand the influence of each element and to verify the potentiality of the thermography for diagnosis. This work focuses on identifying how many parameters can influence the heat transfer mechanism of biological tissues of the neck area and their relation to the detection of a thyroid nodule. For this, combinations of nodular sizes, fat tissue thicknesses, nodule blood perfusion rates, and nodule metabolic heat were analyzed. The protocol for infrared examination used in the university’s hospital was the guide of all studies executed [47,48]. Hereafter, a discussion about the principal results is presented.

Simulations at steady state show that there is a perceptible surface temperature variation between the point just in front of the nodule and its contralateral point for a reduced fat tissue layer. The thermal influence of the nodule’s size and all other parameters on the skin surface decreases when the thickness of the fat layer increases. This was also observed for breast nodules [58].

Almost no skin effect was detected for the thicker fat layer (1.2 cm). Therefore, these simulations show that the thermal insulation effect of the fat tissue is much more important than the nodule size, although, both have the expected influence on surface temperature.

In the transient state, as well as in the steady state, the lower temperature variations were obtained in the skin surface when the thicker fat was considered, showing that this layer reduces the influence of the nodule on the neck’s surface. The larger nodule can be more easily detectable than the smaller nodules. The influence of fat on temperature isolation has also been investigated by Saxena et al. [59], where the presence of stenosis in the carotid artery influences the external skin surface captured and quantified using infrared thermography. Yang and Liu investigated the effects of fat inside the carotid on the human cervical area skin surface temperature [60]. They used the Pennes bioheat equation in a complete 3-D model to characterize the heat transfer in the cervical region [60]. They did an infrared thermal imaging on two persons to evaluate their numerical model, showing that there are variations in skin temperature due to the atherosclerosis plaque in relation to a healthy arterial [60]. They also showed that a dynamic thermal capture (after cooling by alcohol) improves such a detection. This very interesting work reveals the possibility of using infrared imaging as an assessment of atherosclerosis plaque without any wound or radiation to the patients [60]. Their work agrees completely with our observation that fat tissue between thyroid and skin influences on the behavior of temperature measured from infrared sensors.

To analyze the influence of metabolic heat and blood perfusion rate in the simulations, both steady and transient states were evaluated and all combinations of these parameters were considered. For this, a large nodule (with diameters of 3.14 and 1.80 cm) was considered, and at the same time the adipose tissue was neglected (i.e., considered as 0 cm thickness layers, because the inclusion of such layer would only attenuate any difference, in case of its existence). No differences were obtained for any combinations of the metabolic heat and blood perfusion rate performed. Therefore, whatever combination of them, within the possible range is the one representative of malignant or benign nodules: This difference is not possible for detection by skin temperature. However, there is a difference between the temperature series of symmetric points (contralateral regions) on the surface of the skin when the nodule has enough size and there is little fat isolating the nodule. This means that there are conditions enabling to distinguish by infrared sensors where there is a nodule in contralateral position, but disallow one to recognize its classification (benign or malignant).

Simulations at the transient state also show that the final temperatures in the reheating stage are not the same as initial temperatures for all fat thickness considered. This probably shows that five minutes for the reheating stage is not enough to reach the body temperature before neck refrigeration (i.e., the previous stage).

Furthermore, the temperatures of volunteers selected by patients on follow up in the university hospital, acquired by infrared sensor (FLIR camera model SC620) using the simulated protocol [47,48] were analyzed to verify the numerical findings. The temperatures on the neck surface of two patients differing by their BMI, but with nodules of similar dimensions, ultrasound characteristics, and the same diagnostic, show that the temperature changes were higher for a skinny patient as previewed by the numerical simulation.

Other two patients differing as much as possible only by their nodule size, were considered to analyze this influence: Temperatures of the patient with the larger nodule were greater than that with the smaller nodule, agreeing with the numerical simulations about the nodular size influence on skin temperatures. Beyond, two volunteers were selected to have their infrared examinations compared to the identification of the influence of vascularization. Their nodules had the same diagnostic, similar ultrasound characteristics, and their thermography presented the same behavior in the examination. This fact was also observed in the numerical simulations. Finally, the comparison of nodules of the two selected patients with similar characteristics but with different diagnostics, also shows little influence in superficial temperatures. Thus it confirms that there are no relations among the parameters of vascularization and degree of malignancy of the nodules.

## 5. Conclusions

This work studied how several parameters of thyroid nodules (blood perfusion rate, metabolic heat, size, etc.) and the patient body characteristics (thickness of the fat tissue in the neck, for instance) can influence the identification of thyroid nodules by infrared sensors. For this, a bio-heat transfer analysis was performed through numerical simulations by using the COMSOL Multiphysics^®^v5.2 software for Finite Element modeling in order to identify possible relevant parameters. Moreover, infrared examinations of volunteers with nodule diagnostics were analyzed to verify if the numerical results are compatible with reality found in humans.

For simulations, a 2D simplified geometry, based on a consensus image of an average neck, was created. The simulated neck considers skin, fat, and muscle layers, thyroid gland, and an elliptic nodule inside its left lobe. The Pennes’ equation (in both transient and steady state) was used as the mathematical and physical bioheat transfer model. Thermal convection (natural or forced) in the surface of the skin layer, thermal insulation in the trachea boundary surface, and prescribed temperature at 37 ${}^{{}^{\circ}}$C on the remaining surfaces, were considered as boundary conditions. Thermophysical parameters were investigated in the literature for each simulated tissue layer. The importance of thyroid nodule parameters (blood perfusion rate, metabolic heat, size) and body characteristics (thickness of the fat tissue in the neck) were analyzed at transient and steady states. Four fat tissue thickness, three different dimensions of the nodule, and four combinations of blood perfusion and metabolic heat of the nodule were considered in simulation in order to analyze their influence on possible nodule diagnosis.

The principal conclusions can be drawn:

- The simulations showed a great thermal insulation effect of the fat tissue layer. This means that the influence of the heat produced by a thyroid nodule on the surface of the skin will decrease if the layer of fatty tissue is large;
- Simulations showed that the thermal insulation effect of the fat tissue layer is much more important than the nodule size, although, both have the expected influence on surface temperature;
- Inversely, simulations with any combination of metabolic heat and blood perfusion rate of the thyroid nodules could not promote additional difference in temperature on the skin’s surface;
- The analysis of the patient’s infrared examinations agrees with all findings. They show that the fat layer thickness presents great importance on thermal insulating effect;
- Differences in vascular patterns of nodules (with the same diagnostic, similar dimensions, and ultrasonographic characteristics) have no influence on the temperature of the surface during the examination of real patients confirming the results of the numeric study;
- Considering that vascularization could be related to the malignity of the nodules in the thyroid [40], the results indicate difficulties for identification of nodular malignancy by thermography considering equilibrium with an environment of even forced ventilation;
- Although it is possible to identify a thyroidal nodule by using thermography.

## 6. Future Works

For thermography, an immediate consequence of this work is related to the importance of estimating the fat layer in the neck due to its relevance in the analysis and possible association with unfair diagnosis of nodules by IRT. The amount of fat in the neck can be evaluated directly by using US or at least estimated considering the neck perimeter and the BMI of the patients. Moreover, it is relevant to investigate other acquisition protocols to analyze the infrared examinations under all perspectives, in order to present final arguments related to their use (or not) for cancer diagnosis of the thyroid gland (note that here the numerical simulation matches one of them [47,48]).

For the heat simulation aspect, the relation between thermophysical parameters and the biological behavior must be better investigated. They can be measured experimentally in an approach similar to that used for the other tissues of the human body, or they can be estimated through the inverse problem method with thermal examinations of real patients, as well [61]. After, but yet related to simulation: (1) More generic elements and tissue surrounding the neck and their properties (like veins and trachea ventilation) could be included; (2) real geometry of an specific case based, for instance, on magnetic resonance or computer tomography can be modeled; and (3) more nodular elements (shape, size, 3D positions, heterogeneity, and structural constitution) can be included to help in confirming whether the diagnosis of thyroid nodules is really possible by IRT. These could then finally answer any doubt in this research that begins in [30] and to promoting new conclusions simulating the transient behavior.

## Figures and Tables

**Table 1:** Tissue thermophysical parameters used in numerical simulations.

Parameter	Skin	Fat	Muscle	Thyroid	Nodule	Units
Therm. cond.	0.37	0.21	0.47	0.52	0.89	W/(m·K)
Specific mass	1109	911	1090	1050	1050	Kg/m${}^{3}$
Specific heat	3391	2348	3421	3609	3770	J/(Kg·K)
Bl. art. temp.	37.0	37.0	37.0	37.0	37.0	${}^{{}^{\circ}}$C
Bl. perfusion	0.00196	0.000501	0.000708	0.098	0.465	(mL/s)/mL
Metab. heat	1829.85	464.61	1046	4200	42000	W/m${}^{3}$

**Table 2:** Combinations of metabolic heat and blood perfusion for the thyroid nodule used in the simulations. These values are from [26,28,51].

Metabolic Heat (W/m${}^{3}$)	Blood Perfusion ((mL/s)/mL)
4200	0.098
4200	0.465
42,000	0.098
42,000	0.465

**Table 3:** Volunteers identification, diameters of their nodules, nodular ultrasound characteristics (micro-calcification, complete halo, regular contours, heterogeneity, and the Chammas vascularization pattern), cytopathological result using the Bethesda classification, volunteer body characteristics (temperature, mass, height, BMI), and the diagnostic of the nodule.

Patient Id	Diamet.	Calsif.	Halo	Cont.	Het.	Ch.	Beth.	Temp.	Mass	Hgt.	BMI	Diag.
2017-06-12-01	(1.3; 1.1)	No	Yes	Yes	Yes	3	2	36.7	51	155	21.2	B
2016-09-19-01	(1.6; 1.3)	Yes	Yes	Yes	No	2	2	35.5	78	154	32.9	B
2017-10-30-01	(4.2; 2.3)	No	Yes	Yes	Yes	3	2	36.2	62	158	24.8	M
2019-07-03-01	(1.4; 1.0)	No	Yes	Yes	No	3	6	35.0	65	157	26.4	M
2017-09-12-04	(6.7; 3.1)	Yes	Yes	No	No	2	5	36.7	52	154	21.9	M
2019-11-04-03	(6.0; 3.2)	No	Yes	Yes	No	5	3	35.6	76	158	30.4	M
2016-10-31-01	(4.2; 2.6)	No	Yes	Yes	Yes	2	2	35.6	71	172	24.0	B
2018-01-15-01	(3.0; 2.0)	Yes	Yes	Yes	Yes	3	6	36.2	59	155	23.9	M

## Data Availability

All data used in this research were obtained from volunteer patients of the University Hospital at Federal Fluminense University (HUAP/UFF) and are completely available at http://visual.ic.uff.br/en/thyroid/, and accessed on 5 May 2021.
